# Underutilized Medlar (*Mespilus germanica* L.) Fruit as a Source of Dietary Fibers

**DOI:** 10.3390/foods15071222

**Published:** 2026-04-03

**Authors:** Nenad Mićanović, Sanja Stojanović, Aleksandra Margetić, Biljana Dojnov, Jelena Lađarević, Ivana Vukašinović, Jelena Popović-Đorđević

**Affiliations:** 1 University of Belgrade-Faculty of Agriculture, Department of Food Technology and Biochemistry, 11080 Belgrade, Serbia; micanovic.nenad@gmail.com (N.M.); ivanavu@agrif.bg.ac.rs (I.V.); 2 University of Belgrade-Institute of Chemistry, Technology and Metallurgy-National Institute of the Republic of Serbia, Department of Chemistry, 11000 Belgrade, Serbia; sanja.stojanovic@ihtm.bg.ac.rs (S.S.); aleksandra.margetic@ihtm.bg.ac.rs (A.M.); biljana.dojnov@ihtm.bg.ac.rs (B.D.); 3 University of Belgrade, Faculty of Technology and Metallurgy, 11120 Belgrade, Serbia; jmirkovic@tmf.bg.ac.rs

**Keywords:** medlar (*Mespilus germanica* L.) fruit, fruit ripeness, insoluble dietary fibers, enzymatic treatment, fungal enzyme cocktails, FTIR, antioxidant activity, swelling capacity, SEM

## Abstract

Medlar (*Mespilus germanica* L.) is a plant species that belongs to the Rosaceae family. Despite the nutritional and functional value of the medlar fruit, there is limited research, particularly regarding its potential as a source of dietary fibers, indigestible plant-based components, important for improving health. Fungal cellulase enzymes were used to treat medlar fruit in physiological (PRM) and consumable (CRM) maturity and obtain insoluble dietary fibers (IDF). The yield of obtained insoluble dietary fibers was 83% for both PRM and CRM. Fungal strains *Aspergillus welwitschiae* have proven to be significant producers of the cellulase enzyme complex and are also safe for use in food production. Swelling capacity exhibited the most pronounced response to the enzymatic treatment; 8.51–8.65% vs. 12.24–12.86% (untreated and treated fruits, respectively). Dietary fibers extracted from medlar fruits exhibited antioxidant activity that can be attributed to the presence of bound polyphenolic compounds within the fiber material. Microscopic analysis and FTIR spectra revealed structural changes in the medlar fibers due to enzyme activity, indicating partial hydrolysis of lignocellulosic components. This process enhances the functional properties of medlar-based IDF, making it a valuable ingredient for fiber-enriched food products.

## 1. Introduction

Medlar (*Mespilus germanica* L.) is a plant species that belongs to the Rosaceae family and is primarily found in the non-tropical region of the Earth’s northern hemisphere [1,2]. The medlar fruit, which appears after flowering, is round or pear-shaped; yellow–green to dark brown in color; and it has five large seeds and a cup at the top. The fruits are harvested very late, in October and early November. Under storage conditions, the fruits are placed in a dry, cool place until they are over-ripe. During that period, the fruits change color to light brown, while the flesh softens until the fruits are ready for consumption [3]. The fruits are edible throughout the winter because they can be stored in an ordinary warehouse for two to three months, and the fruits can also be found on the branches of the medlar tree until the end of the winter season.

Medlar fruit is a rich source of nutritional and phytochemical compounds. From the nutritional side, medlar fruit includes sugars (glucose, sucrose, fructose, etc.), organic acids (malic, tartaric, oxalic, citric, etc.), vitamins (C, E) [4,5,6,7,8], fatty acids [9,10,11], essential elements [12,13], amino acids, proteins [14,15] and dietary fibers [4,16]. Regarding the phytochemistry of medlar fruit, bioactive compounds, including polyphenols (such as phenolic acids and flavonoids), play a major role in its biological effects [17]. Therefore, medlar should also be considered as a new source of natural polysaccharides, which can be utilized by the food industry as a potential functional ingredient [18].

There are various traditional uses of medlar fruits as a food; it is mostly used fresh [19,20], but food products such as fruit juice, concentrate [21], jam, cheese [22], leather (pestil) [23], and honey [24] can also be found. Moreover, unripe fruits can be used to prepare pickles [22] or beverages such as cider [25]. Data on the chemical composition and biological properties of wild and/or cultivated medlar fruits from the Republic of Serbia are very limited [10,26].

Dietary fiber was initially defined by Trowell as the components of plant cell walls resistant to digestion by human gastrointestinal enzymes [27]. Since then, it has been expanded to include a wider group of nondigestible carbohydrates, as well as other non-carbohydrate compounds such as lignin, that are not digested in the small intestine but may undergo fermentation in the colon [28]. Based on water solubility, dietary fibers are classified as soluble or insoluble dietary fibers. Insoluble dietary fibers include cellulose, hemicellulose, and lignin, while soluble dietary fibers include pectins, gums, and mucilages [29,30]. This classification is important because it relates to their physicochemical properties and their effects on health [31]. Dietary fiber is regarded as essential to a healthy diet, and the World Health Organization (WHO) and European nutritional agencies recommend a daily intake of >25 g. The average European citizen with a daily intake of ∼16–24 g does not reach these recommendations [28,31,32]. Dietary fiber intake provides many health benefits, such as improved digestion and a reduced risk of developing coronary heart disease, stroke, hypertension, diabetes, obesity, and certain gastrointestinal diseases [33,34]. Epidemiological studies regularly find that high dietary fiber intake offers significant health benefits [35]. High fiber consumption has been shown to improve glycemic control, lipid profiles, support weight management, and enhance gut health [33,36,37]. Insoluble dietary fibers are indigestible by human digestive enzymes and must reach the large intestine intact, where they are metabolized by members of the gut microbiota [38]. Insoluble dietary fibers are known to provide broad beneficial clinical outcomes and promote gut homeostasis and general gastrointestinal tract (GIT) health [39]. Insoluble dietary fiber absorbs water and maintains bowel movements, thereby preventing constipation, hemorrhoids, and diverticular disease [38,40]. Soluble dietary fiber increases stomach viscosity and slows digestion, which enhances satiety, decreases caloric intake, and contributes to weight control [41].

Various chemical, biological, and physical methods exist for obtaining or modifying insoluble dietary fiber (IDF), each with specific limitations. Although biological approaches are effective, they are often costly due to the requirement for purified enzymes.

While fungal enzymes have been widely reported to facilitate the release of soluble dietary fibers, their effects on the modification of insoluble dietary fiber (IDF) remain less explored. In the present study, a fungal enzyme complex containing amylase, cellulase, and xylanase was applied to treat medlar in order to modify their fiber structure. These enzymes can act synergistically on lignocellulosic substrates, promoting the degradation and restructuring of the fiber matrix. Similar effects have been reported in previous studies, where the application of comparable enzyme complexes to triticale IDF led to increased porosity and improved adsorption capacity, indicating significant structural modifications within the lignocellulosic network [42].

Given the nutritional composition of medlar (*M. germanica*), its widespread availability, and its underutilization in contemporary diets, it is hypothesized that medlar fruit represents a promising source of insoluble dietary fiber (IDF). In this study, the isolation and characterization of IDF from medlar using fungal enzymatic extract are reported for the first time. This approach enabled the efficient extraction of IDF exhibiting favorable physicochemical properties, thereby supporting its potential application in functional food development and dietary supplementation

## 2. Materials and Methods

### 2.1. Plant Material

**Figure 1:**
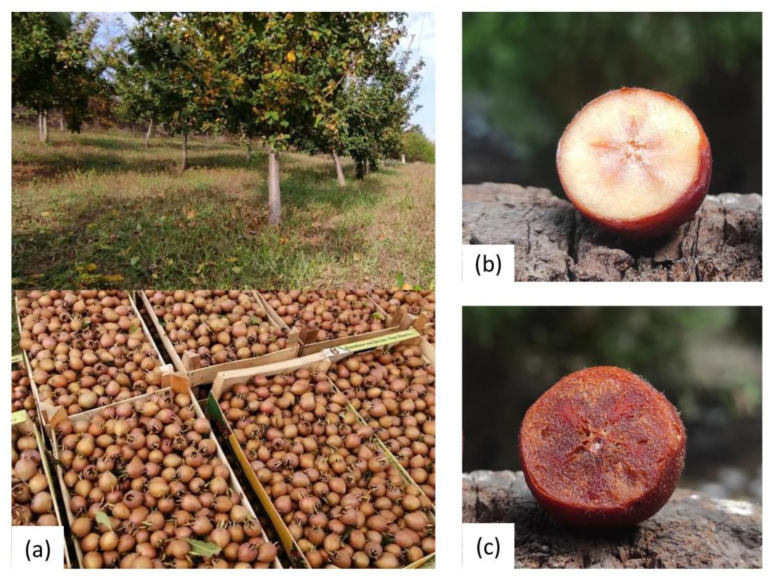
Medlar orchard (**a**) and intersections of physiologically ripe fruit (**b**) and consumable ripe fruit (**c**).

Samples of ‘Domestic medlar’ variety fruit were harvested in November 2023, from a plantation (Figure 1a) near Mali Požarevac (Central Serbia; 49°35′ N; 47°14′ E). Fruits were harvested at the phase of physiological maturity (Figure 1b) and then left in a home cellar to ripen and soften until consumer ripeness (Figure 1c) in an uncontrolled atmosphere, at a temperature of 10 °C for 21 days. Samples of physiologically ripe medlar fruits (PRM) and consumable ripe medlar fruits (CRM) were washed, and seeds were separated from flesh. The skin with the pulp was blended and homogenized, after which the samples were placed in a deep freezer until the lyophilization process.

### 2.2. Microorganisms

Black mold was isolated from various substrates using standard microbiological methods by transferring conidia onto potato dextrose agar (PDA) plates and incubating them at 28 °C for 7 days. After incubation, spores were transferred to fresh PDA to obtain pure cultures, which were then preserved in glycerol stocks and stored at −70 °C in the laboratory strain bank UB483. Once a pure isolate was obtained, strain identification was performed at the genetic level based on the calmodulin (CaM) gene. The results revealed that the strain belongs to *Aspergillus* section *Nigri*, specifically *Aspergillus welwitschiae*, and it was designated as FAW6. The selected FAW6 strain was confirmed to be non-toxigenic through both genetic and analytical analyses. No mycotoxins were detected in the enzyme preparation, and gene cluster analysis showed the absence of biosynthetic pathways responsible for mycotoxin production. This indicates that FAW6 does not produce mycotoxins during cultivation and lacks the genetic potential to do so, making its enzymes safe and suitable for use in fiber processing and the food industry [43].

### 2.3. Production of Fungal Enzyme Complexes (EC)

Strain *A. welwitschiae* FAW6 was cultivated for 96 h on agricultural residues rich in xylan and cellulose (a mixture of corn cob, wheat bran, coarse ground triticale grains) at 28 °C and 60% relative humidity (RH) under solid-state fermentation (SSF) conditions in a thermostat chamber (HPP IPP plus; Memmert USA, LLC; Eagle, WI, USA). After 96 h, the substrates were fully colonized by white mycelium, with no visible sporulation. The fermentation extract used for medlar fruit treatment was obtained by extracting the biomass with 100 mL of 25 mmol/L acetate buffer (pH = 4.5), followed by shaking at 150 rpm for 2 h. The suspension was then centrifuged at 13,257× *g* (Thermo Scientific SL40R; Thermo Fisher Scientific, Waltham, MA, USA) and subsequently microfiltrated using glass microfiber filters (GF/B, 1.0 μm; Whatman, GE Healthcare Life Sciences, Buckinghamshire, UK).

#### Enzyme Activity Assays

Activities of xylanase, amylase, and cellulase were determined using the dinitrosalicylic acid (DNS) method [44]. For the xylanase activity, 10 g/L birchwood xylan served as substrate, and the reaction mixture was incubated at 50 °C for 60 min, with xylose used for calibration [44]. Amylase activity was measured using 10 g/L soluble starch under identical incubation conditions, and maltose was used as the reference standard [42]. Cellulase activity was assayed with 20 g/L carboxymethyl cellulose (CMC) as the substrate under the same reaction conditions, with glucose as the reference standard [45]. One unit of enzyme activity (U/mL) was defined as the amount of enzyme required to release 1 μmol product per minute under the specified assay conditions. All results are presented as the mean of three independent assays ± standard deviation.

### 2.4. Isolation of Insoluble Dietary Fibers from Medlar Fruit

Lyophilized medlar fruit (pulp with skin) samples were used. The samples (PRM and CRM) were subsequently washed with distilled water until a negative reaction to reducing sugars was confirmed using Fehling’s reagent, and then dried at 70 °C prior to analysis. In a 50 mL beaker, 3 g of each medlar sample, PRM, and CRM were added. Four times the volume of fermentation liquid (FL) from the FAW6 strain was added to the fibers. The FL sample of FAW6 was partially purified by gel filtration on a polyacrylamide matrix in order to remove reducing sugars and other small molecules that could be present during the fungal growth on SSF.

**Figure 2:**
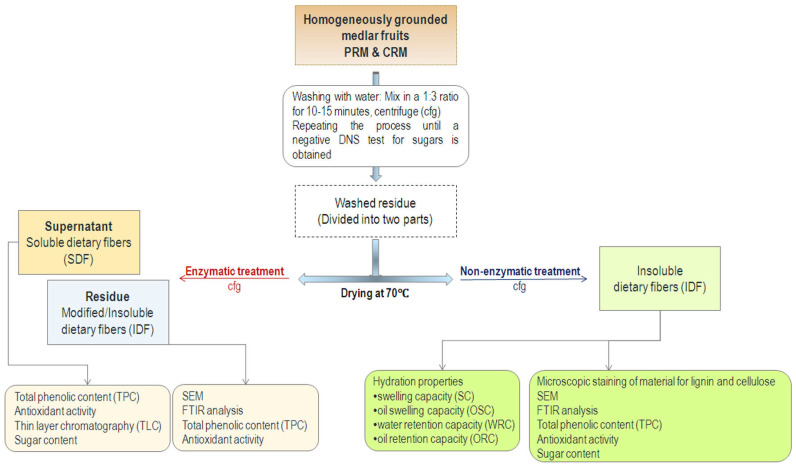
Schematic presentation of the isolation of dietary fibers from physiologically ripe medlar fruits (PRM) and consumable ripe medlar fruits (CRM).

The partially purified enzyme sample used for the enzymatic treatment of medlar contained enzymes from the cellulase and xylanase complex, which play a key role in the breakdown of lignocellulosic material. These enzymes show optimal activity in the temperature range of 37 °C to 50 °C and at a pH of 4.5 to 5.0. Therefore, the reaction was carried out for 25 h at 40 °C and pH 5.0 with constant stirring at 155 rpm. The incubation chamber and all contact surfaces were sterilized according to standard procedures to minimize the risk of microbial contamination. Sterility during the 25 h incubation at 40 °C was ensured by using samples previously dried at 70 °C and an enzymatic complex that was microfiltered prior to use. After 25 h, all samples were centrifuged at 4816× *g* for 20 min at 8 °C. In the obtained supernatants, the presence of carbohydrates and antioxidant capacity were analyzed. The resulting pellets were washed several times with distilled water and dried at 70 °C. The schematic presentation of the isolation of dietary fibers from medlar fruits is presented in Figure 2.

### 2.5. Characterization of IDF

#### 2.5.1. Microscopic Staining of IDF

Structural changes in medlar samples before and after enzymatic treatment were observed using a light microscope (Leitz Diaplan, Wetzlar, Germany) at magnifications of 63× and 250×. For visualization of xylan and cellulose, samples were stained with an aqueous solution containing Safranin-O (28 mmol/L) and Astra Blue (9 mmol/L) at room temperature for 3–5 min, based on literature procedures with slight modifications [46]. Samples were rinsed with water after each staining step, and with ethanol in the final step. After each staining step, samples were rinsed with water, and ethanol was applied in the final step. Prepared samples were mounted on microscope slides and analyzed using a Leitz Orthoplan light microscope (Leitz Diaplan, Wetzlar, Germany) equipped with a Tuscan camera (Tucsen Photonics Co., Ltd., Fuzhou, China) and TSView 7 software.

#### 2.5.2. Examination of the Samples by Scanning Electron Microscope

To describe the morphological properties of the investigated samples, the Scanning Electron Microscope (SEM) method was applied. Surface morphology examination was conducted using a JSM-6390 LV Scanning Electron Microscope (JEOL Ltd., Tokyo, Japan), with an electron energy of 10 KeV and 15 KeV. In order to ensure electrical conductivity, samples were coated with gold in a sputtering chamber SCD 005 Sputter Coater (BAL-TEC GmbH, Schalksmühle, Germany), at 30 mA for 100 s.

#### 2.5.3. ATR-FTIR Analysis

FTIR spectra of both treated and untreated dietary fibers were recorded using a Nicolet™ iS™ 10 FT-IR Spectrometer (Thermo Fisher Scientific), employing the attenuated total reflectance (ATR) technique. The spectra were recorded over the wavenumber range of 4000–500 cm^−1^, with 32 scans collected for each spectrum.

#### 2.5.4. Functional Properties of IDF

The hydration characteristics of dietary fibers (DF) were evaluated through several functional parameters, including swelling capacity (SC), oil swelling capacity (OSC), water retention capacity (WRC), and oil retention capacity (ORC). These parameters were measured at 25 °C following established standardized methods [47].

Furthermore, the hydration properties of insoluble dietary fibers (IDFs) were additionally assessed at 60 °C, considering the known influence of temperature on fiber hydration behavior. Evaluating these properties at elevated temperatures, beyond the standard 25 °C, is particularly important for food processing applications, where thermal treatments are commonly applied. Therefore, assessing hydration at multiple temperatures provides deeper insight into the potential technological performance of the fibers in real food systems.

Functional parameters of insoluble dietary fibers were calculated using the formulas mentioned below:

(1) $$Swelling\;Capacity\;\left(SC\right)=\frac{Volume\;of\;swollen\;fibers\;in\;water\;\left(mL\right)}{Dry\;fibers\;mass\;\left(g\right)}\;$$

(2) $$Oil\;Swelling\;Capacity\;\left(OSC\right)=\frac{Volume\;of\;swollen\;fibers\;in\;oil\;\left(mL\right)}{Dry\;fibers\;mass\;\left(g\right)}$$

(3) $$WRC=\frac{Mass\;of\;fibers\;with\;bound\;water\;\left(g\right)-Dry\;fibers\;mass\;(g)}{Dry\;fibers\;mass\;(g)}\;$$

(4) $$ORC=\frac{Mass\;of\;fibers\;with\;bound\;oil\;\left(g\right)-Dry\;fibers\;mass\;(g)}{Dry\;fibers\;mass\;(g)}\;$$

#### 2.5.5. Measurement of the Total Phenolic Content

Total phenolic content (TPC) was determined by mixing 1 mL of freshly prepared, appropriately diluted Folin–Ciocalteu reagent (1:10) with 0.2 mL of the sample. After 3 min of incubation at room temperature, 0.8 mL 0.7 mol/L Na_2_CO_3_ was added, and the reaction mixtures were kept in the dark for 1 h. Absorbance was measured at 765 nm using a UV-18000 spectrophotometer (Shimadzu, Kyoto, Japan). The experiment was done in triplicate. Quantification was performed using a calibration curve constructed with gallic acid standards, and results were expressed as mg gallic acid equivalents (GAE) per g of sample [42].

### 2.6. Antioxidant Activity of Insoluble Dietary Fibers

#### 2.6.1. ABTS Method

Total antioxidant activity was evaluated using the ABTS^·+^ radical cation assay with minor modification of a previously reported method [48]. The ABTS^·+^ radical was generated by reacting a 7 mmol/L ABTS^·+^ solution with 2.5 mmol/L potassium persulfate (1:1) and allowing the mixture to stand in the dark at room temperature for 12–16 h. Prior to analysis, the solution was diluted with ethanol to achieve an absorbance of 0.70 ± 0.02 at 734 nm. A volume of 0.9 mL ABTS^·+^ working solution was mixed with 0.1 mL of the sample or standard (ascorbic acid), and the absorbance was recorded at 734 nm after 15 min of incubation in the dark. The experiment was done in triplicate. Results were expressed as mg ascorbic acid equivalents per g of dry sample.

#### 2.6.2. DPPH Method

The DPPH^·^ assay was conducted following a reported procedure with slight modifications [49]. A stock solution of DPPH^·^ (1 mmol/L in methanol) was prepared and diluted prior to use to obtain an absorbance of 0.80 ± 0.02 at 515 nm. An aliquot of 0.99 mL of the working DPPH^·^ solution was combined with 10 µL of the sample or Trolox standard (Merck KGaA, Darmstadt, Germany). After incubation in the dark for 15 min, absorbance was measured at 515 nm. The experiment was done in triplicate. Antioxidant capacity was calculated from the calibration curve and expressed as mg Trolox equivalents per g of dry sample.

### 2.7. Analysis of Soluble Carbohydrates After Enzyme Cocktail Treatment

Reducing sugars were quantified using the DNS assay [44], with maltose as the calibration standard. Carbohydrate composition was further analyzed by thin-layer chromatography (TLC) on silica plates (4.5 × 6 cm; Silica gel 60 F-254, Merck, Darmstadt, Germany), using a Camag development chamber (CAMAG, Muttenz, Switzerland). Plates were developed using a double-ascending method in a solvent system consisting of 1-butanol, ethanol, water, and glacial acetic acid (5:3:2:0.5, *v*/*v*/*v*/*v*). Standard oligosaccharide solutions (1.0 mg m/L each) were prepared in water, including glucose (C1), maltose (C2), maltotriose (C3), maltotetraose (C4), maltopentaose (C5), maltohexaose (C6), and maltoheptaose (C7) (Across and Sigma Aldrich, St. Louis, MO, USA). All separations were performed at ambient temperature (22 ± 2 °C). The carbohydrates were detected by spraying the plates with an ethanolic solution containing 5 g/L α-naphthol and 1.02 mol/L H_2_SO_4_, followed by heating for 10 min at 120 °C.

### 2.8. Reagents

All reagents and solvents used in this study were of the highest available purity, purchased from Merck, Sigma-Aldrich, and Qiagen (Hilden, Germany). Agricultural waste materials, triticale (*Triticosecale* sp.), corn cob (*Zea mays* sp.), and wheat bran (*Triticum* sp.) used in SSF for enzyme production were obtained from local producers.

### 2.9. Statistical Analysis

All experiments were performed in triplicate, and the results are presented as mean ± standard deviation (SD). Statistical analyses were carried out using GraphPad Prism 8.4.3 (GraphPad Software, San Diego, CA, USA). One-way analysis of variance (ANOVA), followed by Tukey’s post hoc multiple comparison test, was applied to evaluate differences among produced enzyme activities, as well as among insoluble and soluble dietary fiber (IDF and SDF, respectively) fractions, including their physicochemical characteristics (the hydration properties), antioxidant capacity, and sugar composition. In all cases, differences were considered statistically significant at *p* < 0.05.

## 3. Results and Discussion

### 3.1. Characterization of Enzyme Cocktails

The thesis that fungi that thrive on lignocellulosic material are efficient degraders of lignocellulose has already been proven [50]. Therefore, in this study, the fungal producer of enzyme cocktails (ECs) was cultivated on agro-waste material rich in lignocellulose (corn cob, wheat bran, triticale grains). EC demonstrated significant amylase, xylanase, and cellulase activity, as provided in Table 1.

The strain *Aspergillus welwitschiae* FAW6’s EC had amylase, xylanase, and cellulase activities. Fungi from the genus Aspergillus are known as significant amylase producers on this substrate [51], but they are also known as significant xylanase and cellulase producers [52]. The produced enzymes partially and randomly hydrolyzed lignocellulosic material in IDF, thereby making it more porous.

Cellulases, xylanases, and amylases are hydrolytic enzymes, all classified under glycoside hydrolases, which catalyze the breakdown of glycosidic bonds in various complex carbohydrates. These enzymes play a crucial role in the degradation of plant biomass and starch-rich materials [53]. Cellulases specifically target cellulose, the primary structural component of plant cell walls, breaking it down into glucose units. Similarly, xylanases act on xylan, a major hemicellulose component, releasing xylose and other sugars. Both enzymes are typically present within enzyme complexes and function synergistically in the hydrolysis of lignocellulosic materials, which form a dense, complex matrix with lignin, cellulose, and xylan that limits enzymatic accessibility.

Amylases, on the other hand, hydrolyze starch, a polysaccharide composed of glucose units linked mainly by α-1,4-glycosidic bonds, into simpler sugars such as maltose and glucose. These enzymes are crucial in the digestion and industrial processing of starch-rich substrates, breaking down energy storage polysaccharides rather than structural ones like cellulose or hemicellulose.

The dense, lamellar structure of dietary fibers—consisting of lignin, cellulose, xylan, and other components—poses a significant challenge for enzymatic hydrolysis. As a result, the application of cellulases, xylanases, and amylases to such solid substrates often leads only to partial structural changes. This limited enzymatic accessibility emphasizes the complexity involved in the biotechnological treatment of plant biomass and starch-rich materials [54].

### 3.2. Enzymatic Treatment of Insoluble Dietary Fibers from Medlar

Medlar (*M. germanica*) fruit is a valuable but underutilized source of insoluble dietary fiber (IDF) material, primarily composed of lignocellulosic components such as cellulose, hemicellulose (xylan), and lignin. As indicated in Table 2, a significant portion of the fruit mass remains after enzymatic treatment, suggesting a high content of resistant, fiber-rich material. This lignocellulosic fraction is difficult to degrade, but it contributes to the nutritional value of the fruit, particularly in terms of insoluble fiber content. The economic viability of IDF extraction from medlar is further supported by the relatively high residual mass yield. Despite the short postharvest shelf life of medlar and its seasonal availability, utilizing this fruit for fiber extraction is a sustainable strategy to add value to an otherwise underused resource.

### 3.3. Microscopic Analyses of Obtained IDF

**Figure 3:**
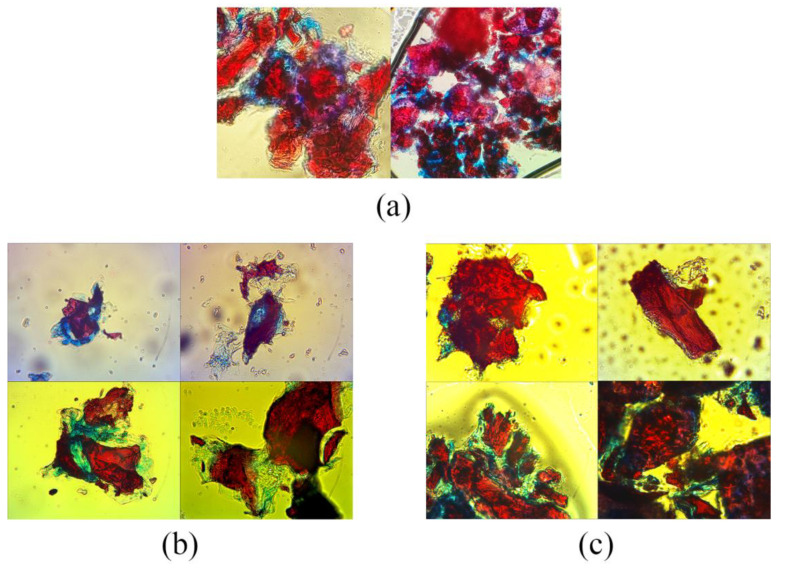
Microscopic view after staining of the medlar (*M. germanica*) fruit samples: (**a**) untreated IDF, (**b**) physiologically ripe medlar (EPRM) fruits treated with the enzymatic complex FAW6, (**c**) consumable ripe medlar (ECRM) fruits treated with the enzymatic complex FAW6. Safranin-O dyes lignin red. Astra blue dyes cellulose blue.

Microscopic analysis revealed distinct structural differences between untreated (Figure 3a) and enzymatically treated (Figure 3b,c) medlar fruit samples, both in physiologically ripeness and consumable ripeness stages. Treatment with the FAW6 enzymatic complex led to evident changes in the organization and integrity of the fibrous tissues. Samples treated with the FAW6 complex (Figure 3b,c) exhibited reduced staining intensity in these regions, suggesting partial hydrolysis of cellulose and hemicellulose. The observed degradation of the plant cell wall is a consequence of the enzymatic activity of cellulases and xylanases from the FAW6 complex, which catalyze the breakdown of lignocellulosic substrates in the plant cell wall.

These structural modifications are consistent across both unripe and ripe medlar tissues, indicating that the enzymatic complex is effective regardless of the developmental stage of the fruit. The partial degradation of cell wall components implies an increase in porosity and a weakening of the rigid polysaccharide structure, which, in turn, may enhance the mobility and interaction of water and oil within the fibrous material.

The microstructural changes observed microscopically correspond well with the results from functional property measurements, including swelling capacity, oil swelling, water retention capacity, and oil retention capacity. Since these parameters are directly influenced by the material’s porosity and surface properties, the enzymatic disruption of cellulose and hemicellulose appears to facilitate greater fluid absorption and retention. In particular, the reduction in cellulose crystallinity and the partial solubilization of hemicelluloses likely contribute to increased hydrophilicity and the ability of the matrix to retain oil and water [42,50].

Overall, the results demonstrate that enzymatic treatment with FAW6 not only induces visible microstructural changes in medlar fruit tissues but also significantly impacts their functional characteristics. These findings are important for applications where the enhancement of adsorption or retention capacity is desired, such as in food processing or dietary fiber modification.

In untreated samples, the fibrous structure appeared dense and intact, with cellulose-rich regions distinctly stained blue, indicating the presence of undegraded polysaccharide components. In contrast, samples treated with the FAW6 complex (Figure 3b,c) exhibited reduced staining intensity in these regions, suggesting partial hydrolysis of cellulose and hemicellulose. The observed degradation of the plant cell wall is a consequence of the enzymatic activity of cellulases and xylanases from the FAW6 complex, which catalyze the breakdown of lignocellulosic substrates in the plant cell wall [42].

**Figure 4:**
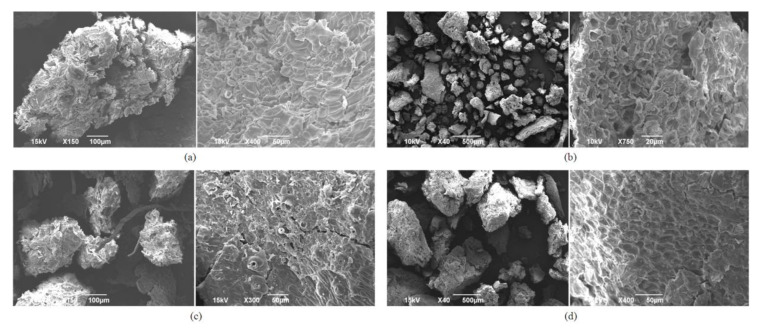
Surface morphology of: (**a**) untreated physiologically ripe medlar (PRM), (**b**) physiologically ripe medlar treated with the enzymatic complex FAW6 (EPRM), (**c**) untreated consumable ripe medlar (CRM), and (**d**) consumable ripe medlar fruits treated with the enzymatic complex FAW6 (ECRM).

Additionally, SEM analysis of the surfaces of the obtained fibers showed that medlar fibers have a large surface area, which promises high adsorption capacity and significant properties. Figure 4a shows an untreated physiologically ripe medlar (PRM)—typical aggregate, which appears to have a length greater than 500 μm. Dimensions of untreated consumable ripe medlar (CRM) aggregates (Figure 4c) at the same 150× magnification are smaller than the PRM sample and appear to be in the 100 μm to 300 μm range. Differences in the surfaces of enzymatically treated fibers, both PRM (Figure 4b) and CRP (Figure 4d), compared to the same untreated ones (Figure 4a,c), are more noticeable and unequivocally confirm that there have been changes resulting in an increased surface area.

These structural modifications are consistent across both physiologically and consumable medlar tissues, indicating that the enzymatic complex is effective regardless of the developmental stage of the fruit. The partial degradation of cell wall components implies an increase in porosity and a weakening of the rigid polysaccharide structure, which, in turn, may enhance the mobility and interaction of water and oil within the fibrous material. The observed microstructural changes correspond well with results from functional property measurements, including swelling capacity, oil swelling, water retention capacity (WRC), and oil retention capacity (ORC). Since these parameters are directly influenced by the material’s porosity and surface properties, the enzymatic disruption of cellulose and hemicellulose appears to facilitate greater fluid absorption and retention. In particular, the reduction in cellulose crystallinity and the partial solubilization of hemicelluloses likely contribute to increased hydrophilicity and the ability of the matrix to retain oil and water [42].

Overall, the results of microscopic analysis and SEM demonstrate that enzymatic treatment with FAW6 not only induces visible microstructural changes in medlar fruit tissues but also significantly impacts their functional characteristics. These findings are important for applications where the enhancement of adsorption or retention capacity is desired, such as in food processing or dietary fiber modification.

### 3.4. FTIR Spectra of Medlar Fruit Samples

**Figure 5:**
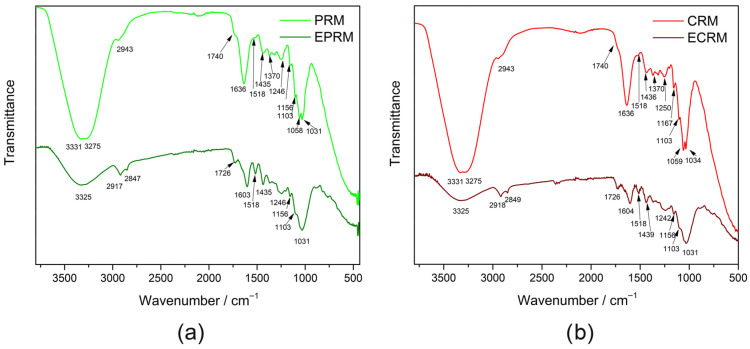
FTIR spectra of (**a**) non-treated and enzyme-treated physiologically ripe fruit (PRM and EPRM, respectively), and (**b**) non-treated and enzyme-treated consumable ripe fruit (CRM and ECRM, respectively).

The FTIR spectra of PRM and CRM (Figure 5a,b) show characteristic absorption bands corresponding to the main biochemical components of the medlar fruit [4]. A broad band appears around 3300 cm^−1^, originating from O–H stretching vibrations of hydroxyl present in polysaccharides and phenolic compounds. The absorption at 2943 cm^−1^ is assigned to C–H stretching vibrations of methyl and methylene groups found in lipids, cellulose, and other aliphatic compounds. A distinct band at 1740 cm^−1^ is assigned to C=O stretching vibrations of pectin and hemicellulose ester group [4,55]. The broad band observed at 1636 cm^−1^ arises mainly from the stretching vibrations of C=O in carbonyl compounds and C=C stretching vibrations of aromatic compounds. Also, this peak may appear from the bending vibration of absorbed water molecules and the carboxyl group of pectin [4]. A shoulder at 1518 cm^−1^ appears from the C=C stretching vibrations of aromatic compounds present in medlar. The absorption at 1435 cm^−1^ in PRM and 1436 cm^−1^ in CRM originates from C–H symmetric bending of –CH_2_ characteristic of cellulose [55], and carboxylic group in pectin [4]. The 1370 cm^−1^ band is assigned to symmetric C–H bending of methyl groups coupled with O–H bending modes in polysaccharides [56]. The peak at 1317 cm^−1^ represents C–H bending vibrations associated with –CH_2_ groups. The well-defined band 1246 cm^−1^ in PRM and 1250 cm^−1^ in CRM arise from C–O–C asymmetric stretching and C–O stretching vibrations of ester groups and polysaccharides, mainly pectin and hemicellulose [56]. At lower wavenumbers (1100–1000 cm^−1^), intense absorption bands are observed, corresponding to C–O–C and C–O stretching vibrations.

**Figure 6:**
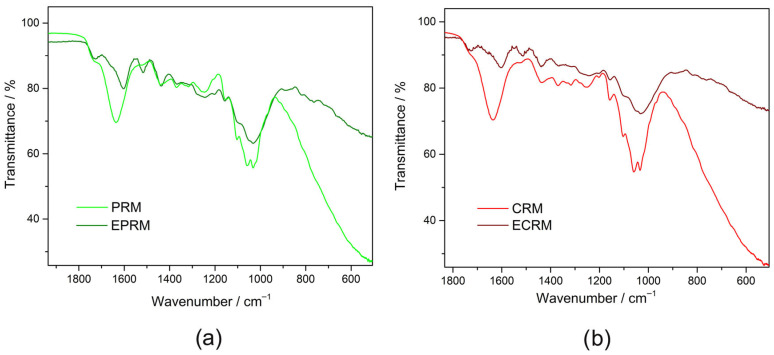
FTIR spectra in the ‘fingerprint’ region of: (**a**) physiologically ripe fruit (PRM), (**b**) consumable ripe fruit (CRM), and relevant fruits after the treatment with enzymes (EPRM and ECRM, respectively).

Following enzymatic treatment, the FTIR spectra of EPRM and ECRM (Figure 5a,b) exhibited distinct modifications in both intensity and band position, confirming the hydrolytic action of the applied enzymes. The O–H stretching band exhibited a strong reduction, becoming blunted and broader, which indicates reorganization of hydroxyl-bearing compounds and weakening of hydrogen bonding due to the cleavage of hydroxyl-rich polysaccharides. The C–H stretching region (2917–2849 cm^−1^) displayed sharper and more intense peaks, reflecting disruption of aliphatic structures in cellulose and hemicellulose. The ester carbonyl band at 1740 cm^−1^ shifted to 1726 cm^−1^ becoming sharper, while the band at 1636 cm^−1^, in both PRM and CRM, diminished with the emergence of a peak at 1603 cm^−1^ in PRM, and 1604 cm^−1^ in CRM. The band at 1518 cm^−1^ became sharper and intensified. In the ‘fingerprint’ region (Figure 6), the characteristic carbohydrate bands between 1370 and 1240 cm^−1^ changed intensities and shapes, indicating changes in the cellulose and hemicellulose structural order. The region between 1100 and 1000 cm^−1^ associated with C–O–C and C–O stretching vibrations becomes dominant and blunted in the spectra of EPRM and ECRM due to the accumulation of soluble mono- and oligosaccharides in medlar samples by enzymatic degradation of cellulose and hemicellulose.

Enzymatically treated samples showed subtle changes in band intensity and shape, particularly in regions associated with hydrogen bonding and glycosidic linkages. These modifications suggest partial disruption of intermolecular interactions without extensive degradation of the polysaccharide framework. The reduced intensity and slight broadening of hydroxyl-related bands are consistent with increased molecular mobility and enhanced water accessibility within the fiber structure. Such spectral changes correlate well with the observed increase in swelling capacity, supporting the hypothesis that enzymatic treatment primarily improves hydration behavior through microstructural rearrangement rather than chemical transformation. The observed reduction in hydrogen bonding intensity correlates with increased swelling capacity, suggesting improved water accessibility within the fiber matrix.

### 3.5. Characterization of Insoluble Dietary Fibers

The residual fibrous material, predominantly composed of insoluble fibers such as lignin-rich fragments and non-degraded cellulose, remains present in the treated medlar samples. This insoluble fraction retains functional benefits related to mechanical bulking, water-holding capacity, and gradual fermentation in the colon. Thus, enzymatic treatment appears to enable dual functionality: (1) enhancing the structural and adsorption properties of the residual fiber material, and (2) releasing soluble bioactive components with potential health benefits.

The functional properties of insoluble dietary fiber fractions obtained from untreated (PRM, CRM) and enzyme-treated samples (EPRM, ECRM) are presented in Table 3. The water retention capacity (WRC) at 25 °C differed slightly among the samples, with CRM and EPRM showing significantly higher values than PRM and ECRM (*p* < 0.05). At 60 °C, untreated samples (PRM and CRM) exhibited significantly higher WRC values compared to enzyme-treated samples, while ECRM showed the lowest value (*p* < 0.05). Oil retention capacity (ORC) at 25 °C showed minor variations, whereas enzyme-treated samples exhibited slightly higher values. PRM did not differ significantly from either group. In contrast, no significant differences in ORC were observed among the samples at 60 °C (*p* > 0.05). Among the evaluated parameters, swelling capacity (SC) exhibited the most pronounced response to enzymatic treatment. Both enzyme-treated samples showed significantly higher SC values compared to the untreated fibers (*p* < 0.05), with ECRM displaying the highest value. A similar trend was observed for oil swelling capacity (OSC), where enzymatic treatment significantly increased OSC compared to untreated samples (*p* < 0.05). The increase in SC suggests that enzymatic modification promoted structural loosening of the fiber matrix, enabling greater water penetration and volumetric expansion. In contrast, changes in WRC and ORC were less pronounced, indicating that enzymatic treatment primarily affected fiber porosity rather than the availability of specific hydrophilic or lipophilic binding sites.

This distinction is technologically relevant, as swelling capacity is closely associated with physiological effects such as satiety enhancement and intestinal bulking, while WRC and ORC are more strongly influenced by surface chemistry. The observed predominance of SC over WRC improvement implies that the applied enzyme cocktail facilitated internal structural rearrangements rather than extensive depolymerization.

Importantly, the absence of functional differences between PRM and CRM fibers demonstrates that insoluble dietary fiber with comparable technological performance can be obtained prior to full edible ripening. This finding represents a practical advantage, as early-stage processing reduces postharvest losses and shortens storage requirements.

Dietary fibers have significant application in food technology, as they are more and more used as functional food ingredients [57,58]. Due to their technological properties, such as water and oil retention, they can be used to maintain and extend the freshness of products such as bakery products, meat, and beverages [59]. Dietary fibers have also found application in stabilizing emulsions and improving the textural properties of food products. They can also be used as a substitute for fats and oils [60]. Although emulsifying properties were not directly evaluated in this study, the determined functional characteristics (WRC, ORC, SC, and OSC) provide useful insight into the interaction of dietary fiber with water and lipid phases in food systems. These properties may indirectly contribute to the stabilization of complex food matrices, including emulsion-based products, through mechanisms such as water binding, oil retention, and increased viscosity. Dietary fibers also enrich the nutritional profile and increase the nutritional quality in processed foods [57]. Since dietary fiber is a component found in large quantities in by-products of the fruit and grain processing industry, such as peels, pomace, and husks, which are commonly used as animal feed and fertilizers, it has the potential to be converted into product value. There is interest in exploiting the potential of using health-beneficial components and other valuable ingredients of fruit and grain by-products in the development of functional foods in order to increase the utilization of food industry by-products [58].

### 3.6. Total Phenolic Content and Antioxidant Activity of Dietary Fibers

Dietary fiber, in its native form, is associated with naturally occurring phenolic compounds, carotenoids, and other minor constituents that have antioxidant properties. Such fiber is classified as “antioxidant dietary fiber” (ADF). Antioxidant dietary fiber can provide a synergistic antioxidant capacity that helps protect against oxidative stress. Dietary fiber contributes to antioxidant properties primarily because it acts as a carrier for phenolic compounds and other antioxidant compounds found in plant-based foods [61]. Consumers are increasingly looking for natural food-based strategies for disease prevention. In that respect, the combination of dietary fiber and polyphenol content has become a critical parameter in the design and evaluation of functional food ingredients and dietary supplements [62].

The antioxidant potential of dietary fibers represents one of their most valuable functional attributes, particularly in the development of functional foods aimed at promoting health and preventing chronic diseases [61]. The phenolic compounds, often associated with the fiber matrix, contribute significantly to the overall antioxidant activity by scavenging free radicals and lowering oxidative stress, which is a key factor in the development of cardiovascular diseases, type 2 diabetes, and certain cancers [61,63]. Cereal fibers, such as those from wheat, oats, and barley, are rich in ferulic acid and other phenolic acids [63,64], while medlar fibers contain flavonoids and other bioactive polyphenols with demonstrated antioxidant effects [26,65]. The incorporation of such fiber sources into food products can enhance their functional value, not only through traditional physiological benefits (e.g., improved gut health and metabolic regulation), but also through the delivery of antioxidant protection. 

The results indicate that dietary fibers extracted from medlar fruit exhibit notable antioxidant activity (Table 4), which can be attributed to the presence of bound polyphenolic compounds within the fiber material. This enhancement may be attributed to partial cell wall disruption, which facilitates the release or exposure of phenolic compounds previously entrapped within the polysaccharide network. The total phenolic content (TPC) of soluble dietary fiber increased after enzyme treatment, while the content of insoluble dietary fiber slightly decreased, due to the enzymes breaking down structural carbohydrates and cell wall components, releasing phenolic compounds that were previously bound to the insoluble matrix. This disruption increases the solubility and extractability of phenolics into the soluble fraction while reducing the amount remaining in the insoluble fraction [66]. On the other hand, non-extractable antioxidants bound to dietary fibers can have their activity restored by extractable antioxidants, which donate electrons or hydrogen atoms to regenerate. During digestion, these compounds can neutralize free radicals while being continuously regenerated [67]. Bound phenolics are largely resistant to digestion in the upper gastrointestinal tract and reach the colon intact, where they are released through microbial fermentation. Once liberated, these compounds are extensively metabolized by the gut microbiota into smaller phenolic metabolites that may exert local anti-inflammatory and antioxidant effects, as well as modulate microbial composition and activity [68,69]. Therefore, the primary significance of phenolic binding to insoluble fiber lies not in its direct antioxidant capacity, but in its role as a delivery system that enables the gradual release of bioactive compounds in the colon and promotes microbiota-mediated health effects [70,71]. The increase in antioxidant activity was observed for both PRM and CRM samples, with no statistically significant differences between ripening stages. These results suggest that medlar’s insoluble dietary fiber may serve as a carrier of bioactive compounds, contributing to potential health benefits beyond basic dietary fiber functionality. These findings are consistent with previous studies highlighting the role of flavonoids and other bioactive compounds identified in medlar [26,65]. The observed antioxidant capacity supports the functional value of these fibers beyond their physiological effects, suggesting potential for application in functional food formulations.

In particular, the antioxidant properties may contribute to reducing oxidative stress in the human body, which is linked to the prevention of chronic diseases such as cardiovascular conditions, diabetes, and certain cancers [61,63]. Therefore, the integration of such fiber-rich sources into the human diet may serve not only as a source of dietary fiber but also as a carrier of natural antioxidants. This dual functionality reinforces the relevance of antioxidant capacity as a key quality indicator for fiber-based food ingredients, and aligns with the growing interest in functional foods that provide added health benefits.

### 3.7. Analysis of Soluble Carbohydrates Obtained from IDF After EC Treatment

**Figure 7:**
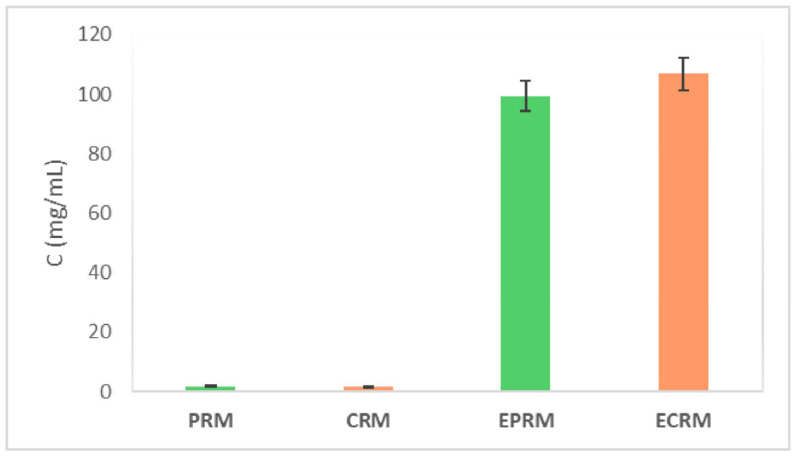
Concentration of reducing sugars in soluble fibers from physiologically ripe fruit and consumable ripe medlar fruit (PRM and CRM, respectively), and after enzymatic treatment (EPRM and ECRM). Results are presented as mean ± SD (*n* = 3).

The analysis of soluble sugar in the supernatants collected after EC treatment of medlar IDF indicates enzymatic hydrolysis of cellulose, xylan, and residual starch remaining from the IDF isolation process. Quantification of reducing sugars using the DNS assay revealed significant differences in the concentrations of released sugars between enzymatically treated IDF and untreated samples, as presented in Figure 7.

**Figure 8:**
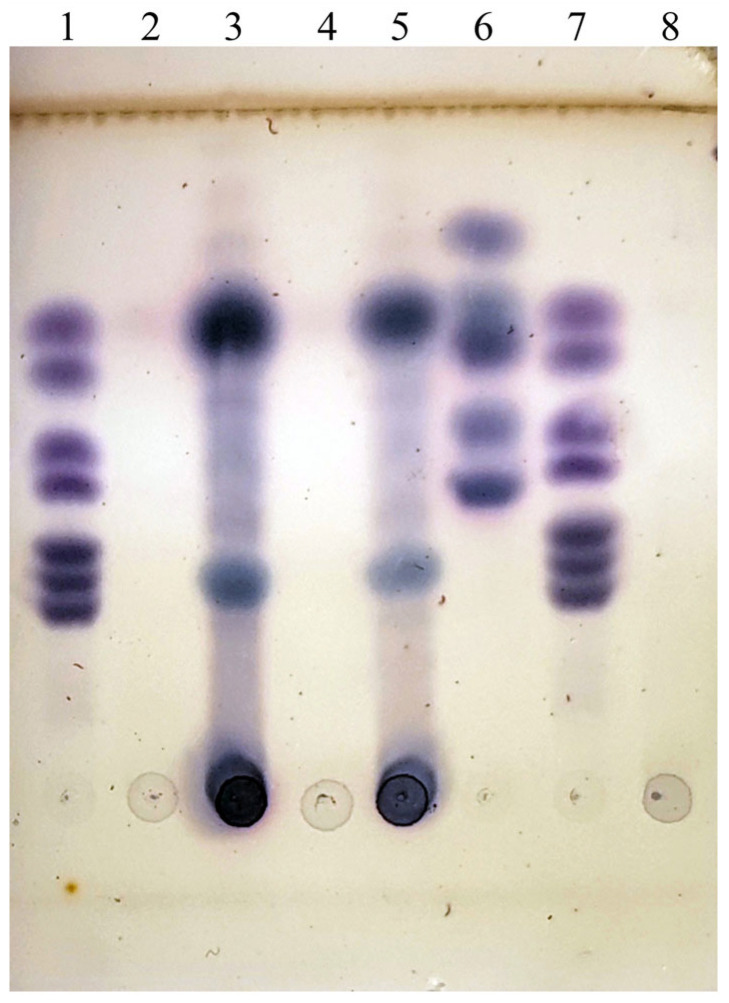
TLC analysis of soluble fibers from medlar fruit after enzymatic treatment. The order of samples: 1. Standards 1–7 β-glucose, 2. PRM—Untreated physiologically ripe fruit, 3. EPRM—Enzymatically treated physiologically ripe fruit, 4. CRM—Untreated consumable ripe fruit, 5. ECRM—Enzymatically treated consumable ripe fruit, 6. XOS standards (1–4) + arabinose, 7. Standards 1–7 β-glucose, 8. Partially purified enzymatic complex FAW6.

The results obtained from the thin-layer chromatography (TLC) analysis (Figure 8) provided further evidence of the biochemical transformations occurring during the enzymatic treatment of medlar samples. Notably, the appearance of specific sugar bands in the enzymatically treated samples (samples 3 and 5) indicates the release of low molecular weight carbohydrates—primarily monosaccharides and oligosaccharides—which are absent in the untreated control samples (samples 2 and 4). Sample 8, which represents the FAW6 enzymatic complex alone, showed no visible sugar bands, confirming that the observed sugars originated from the enzymatic degradation of the medlar substrates, and not from residual sugars in the enzyme preparation itself.

The presence of oligosaccharides, which are recognized as soluble dietary fibers, is of particular interest. These compounds are released as a result of the enzymatic cleavage of hemicellulose and partially hydrolyzed cellulose chains, processes catalyzed by xylanase and cellulase enzymes in the FAW6 complex. Soluble fibers, such as oligosaccharides, are known for their prebiotic potential, promoting beneficial gut microbiota, and are often associated with improved gastrointestinal health and systemic metabolic effects [72].

While the primary purpose of enzymatic treatment may be to alter structural or functional properties of the plant material (e.g., for better swelling or adsorption capacity), the production of soluble sugars represents an added-value co-product. These compounds could be further isolated, purified, and utilized as functional food ingredients, prebiotic supplements, or fermentation substrates in various industrial applications [73].

## 4. Conclusions

Insoluble dietary fibers have been isolated from medlar fruit for the first time, addressing its current underutilization in human nutrition. The extraction yield was notably high, indicating that a substantial proportion of the fruit consists of IDF, which supports the economic feasibility of its valorization. Based on their physicochemical and bioactive properties, the isolated fibers demonstrated potential as functional additives in different food formulations. The enzymatic extraction approach, employing fungal enzyme complexes, proves effective, yielding IDF with enhanced swelling and oil-holding capacities, and antioxidant potential attributes that further reinforce their applicability in functional food development. These findings reinforce the multifaceted advantages of enzyme-assisted modification, offering potential for both functional and nutritional innovation in the development of value-added products from fruit by-products or less-utilized plant material.

The absence of significant differences between physiologically ripe and consummably ripe fruits represents a technological advantage, allowing fiber extraction prior to full ripening and reducing losses due to spoilage. The lack of significant differences between fibers obtained from physiologically ripe and consumable ripe fruits indicated that early-stage processing is feasible, offering technological and economic advantages by reducing storage time and postharvest losses. Structural analyses confirmed that functional improvements were primarily associated with increased porosity and hydration capacity rather than chemical degradation. Given its limited presence in the human diet, promoting the use of medlar as a source of dietary fiber could help diversify fiber sources and reduce waste from seasonal fruit surpluses.

## Figures and Tables

**Table 1:** Enzymatic activities of enzyme cocktails (EC) used in the treatment of IDF.

	Amylase Activity (U/mL)	Cellulase Activity (U/mL)	Xylanase Activity (U/mL)
FAW6	8.6 ± 0.3	9.5 ± 0.4	29 ± 1

Values are presented as mean ± SD (*n* = 3).

**Table 2:** Yield of obtained insoluble dietary fibers.

Sample	Mass of IDF ^1^ Before Enzymatic Treatment (g)	Volume of Added Fermentation Liquid (mL)	Mass of IDF After Enzymatic Treatment (g)	Yield (%)
PRM	3.0 ± 0.1 ^2^	12.0 ± 0.4	2.5 ± 0.1	83 ± 4
CRM	3.0 ± 0.1	12.0 ± 0.5	2.5 ± 0.1	83 ± 4

^1^ IDF—Insoluble dietary fibers; ^2^ Values are presented as mean ± SD (*n* = 3).

**Table 3:** Hydration properties of untreated and enzyme-treated medlar fruits.

IDF Sample	WRC ^1^ (g/g)		ORC (g/g)		SC (mL/g)	OSC (mL/g)
	**25 °C**	**60 °C**	**25 °C**	**60 °C**	**r.t. ^2^**	**r.t.**
PRM	2.53 ± 0.06 ^a3^	3.72 ± 0.04 ^a^	1.04 ± 0.02 ^a,b^	0.86 ± 0.04 ^a^	8.64 ± 0.2 ^a^	2.50 ± 0.02 ^a^
CRM	2.79 ± 0.03 ^b^	3.72 ± 0.02 ^a^	0.96 ± 0.06 ^a^	0.95 ± 0.04 ^a^	8.74 ± 0.1 ^a^	2.50 ± 0.03 ^a^
EPRM	2.73 ± 0.08 ^b^	2.84 ± 0.06 ^b^	1.08 ± 0.04 ^b^	0.86 ± 0.03 ^a^	12.26 ± 0.0 ^b^	2.81 ± 0.01 ^b^
ECRM	2.48 ± 0.03 ^a^	2.62 ± 0.04 ^c^	1.08 ± 0.04 ^b^	0.96 ± 0.06 ^a^	12.86 ± 0.0 ^c^	2.70 ± 0.01 ^c^

^1^ WCR—water retention capacity; ORC—oil retention capacity; swelling capacity (SC), oil swelling capacity (OSC); ^2^ r.t.—room temperature; ^3^ Values are presented as mean ± SD. Values sharing the same letter are not significantly different (*p* < 0.05) (*n* = 3).

**Table 4:** Total phenolic content and antioxidant activity of soluble and insoluble dietary fibers from untreated and enzyme-treated medlar fruits.

IDF Sample	TPC (mg/mL)	ABTS^·+^ (µmol/mL AE) ^1^	DPPH^·^ (mmol/mL TE)
Soluble dietary fibers			
PRM	19.6 ± 0.3 ^a2^	82.7 ± 0.5 ^a^	0.62 ± 0.02 ^a,c^
EPRM	74.8 ± 0.4 ^b^	62.4 ± 0.3 ^b^	0.72 ± 0.02 ^b,e^
CRM	18.5 ± 0.4 ^a^	83.7 ± 0.4 ^a^	0.60 ± 0.02 ^a^
ECRM	68.0 ± 3.0 ^c^	67.4 ± 0.2 ^c^	0.67 ± 0.01 ^c,e^
FT FAW6	29.4 ± 0.5 ^d^	75.5 ± 0.4 ^d^	0.38 ± 0.01 ^d^
Insoluble dietary fibers			
PRM	10.1 ± 0.3 ^a^	61.2 ± 0.3 ^a^	2.52 ± 0.01 ^a^
EPRM	9.4 ± 0.3 ^b^	62.5 ± 0.3 ^b^	2.80 ± 0.01 ^b^
CRM	18.3 ± 0.5 ^a^	68.1 ± 0.2 ^c^	2.26 ± 0.02 ^c^
ECRM	16.5 ± 0.4 ^c^	53.7 ± 0.3 ^d^	3.35 ± 0.01 ^d^

^1^ mg/mL—milligrams per milliliter; µmol/mL AE—micromoles per milliliter Ascorbic Acid Equivalents; mmol/mL TE—millimoles per milliliter Trolox Equivalents; ^2^ Values are presented as mean ± SD (*n* = 3); Values sharing the same letter are not significantly different (*p* < 0.05).

## Data Availability

The original contributions presented in this study are included in the article. Further inquiries can be directed to the corresponding author.

## Abbreviations

The following abbreviations are used in this manuscript:

ABTS^·+^	2,2′-Azino-bis(3-ethylbenzothiazoline-6-sulfonic acid)
ADF	Antioxidant dietary fiber
ATR-FTIR	Attenuated total reflectance—Fourier Transform Infrared Spectroscopy
CaM	Calmodulin gene
CMC	Carboxymethyl cellulose
CRM	Consumable ripe medlar fruit
DF	Dietary fibers
DNS	Dinitrosalicylic acid
DPPH^·^	2,2-Diphenyl-1-picrylhydrazyl
EC	Enzyme cocktails
ECRM	Consumable ripe medlar fruits treated with the enzymatic complex FAW6
EPRM	Physiologically ripe medlar fruits treated with the enzymatic complex FAW6
FL	Fermentation liquid
IDF	Insoluble dietary fibers
ORC	Oil retention capacity
OSC	Oil swelling capacity
PDA	Potato dextrose agar
PRM	Physiologically ripe medlar fruit
RH	Relative humidity
SC	Swelling capacity
SDF	Soluble dietary fiber
SEM	Scanning Electron Microscope
SSF	Solid-state fermentation
TLC	Thin-layer chromatography
TPC	Total Phenolic Content
WRC	Water retention capacity
XOS	Xylooligosaccharides
